# Nitrogen and phosphorus co‐limitation of tree growth in northern hardwood forests

**DOI:** 10.1002/ecy.70217

**Published:** 2025-10-07

**Authors:** Noah M. Blumenthal, M. Henry H. Stevens, Shinjini Goswami, Ruth D. Yanai, Timothy J. Fahey, Melany C. Fisk

**Affiliations:** ^1^ Biology Department Miami University Oxford Ohio USA; ^2^ School of Natural and Health Sciences Lees‐McRae College Banner Elk North Carolina USA; ^3^ Department of Forest and Natural Resource Management SUNY College of Environmental Science and Forestry Syracuse New York USA; ^4^ Department of Natural Resources Cornell University Ithaca New York USA

**Keywords:** American beech, basal area increment, birch, co‐limitation, MELNHE, nutrient fertilization, secondary succession, sugar maple

## Abstract

Nutrient limitation of forest growth has been difficult to predict, and in temperate forests, long‐term tests of single‐nutrient versus multiple‐element limitation are few. Nutrient co‐limitation is the expected outcome of the ability of plants to adjust allocation to minimize limitation by any single resource. Nutrient limitation of productivity in northern hardwood forests was predicted by the Multiple Element Limitation (MEL) model to shift over time since harvest from single limitation by N to P at ~30 years and then, in mature forests, to co‐limitation by N and P. Our work tested those predictions for tree growth in a fully factorial N and P addition experiment in 13 forest stands that we grouped in young (20–30 years), mid‐age (40–50 years), and mature (>100 years old) age classes in New Hampshire, USA. Over 8 years of treatment, we found evidence of additive co‐limitation of tree growth by N and P. We did not find evidence that limitation varied with time since disturbance. Our results suggest that processes contributing to co‐limitation in these northern hardwood forests are effective across stands that vary widely in N status and are not sensitive to disturbance by forest harvest over time periods of several decades.

## INTRODUCTION

Ecosystem theory based on soil development predicts single‐nutrient limitation of productivity. Limitation by nitrogen (N) on geologically young soils and by phosphorus (P) on older soils is expected because N is scarce in soil parent materials but is continuously available from atmospheric N_2_ fixation, whereas mineral P is sourced from parent materials but is occluded or lost as weathering progresses. These predictions have been supported in chronosequences of soil development (Harrington et al., 2001; Walker & Syers, 1976; Wardle et al., 2004) and in a variety of manipulation studies in temperate and tropical forests (e.g., Hedin, 2004; Hou et al., 2012; Li et al., 2018; Vadeboncoeur, 2010). However, predictions about ecosystem productivity based on soil age and development are not always supported. For instance, P limitation has been found on young soils following glacial retreat (Darcy et al., 2018), in spruce forest in Scandinavia (Almeida et al., 2019), and in northern hardwoods in our study sites in the northeastern United States (Goswami et al., 2018). Likewise, N limitation has been found on older soils of lowland tropical forests in Costa Rica (Alvarez‐Clare et al., 2013). Meta‐analyses have reported a variety of findings, including widespread N limitation regardless of soil age and ecosystem development in temperate and tropical systems (LeBauer & Treseder, 2008), both N and P limitation in lowland tropical forests (Wright et al., 2018), and the importance of parent material to P limitation on soils of any age (Augusto et al., 2017).

That simple generalizations about nutrient limitation have proven difficult to support is not surprising given controls of nutrient availability by multiple interacting mechanisms other than soil age and ecosystem development. Which nutrient is most limiting on either a young or old soil can also depend on influences of disturbance or anthropogenic change, or on biotic processes such as N fixation and the balance between plant and microbial demands (Vitousek et al., 2010; Vitousek & Field, 1999). Furthermore, various processes whereby plants and soil organisms modify the supply or availability of a limiting nutrient can mitigate single‐nutrient limitation and produce co‐limitation (i.e., coincident limitation by more than one nutrient), if those processes are sufficient to balance the acquisition of different nutrients and minimize limitation by any single resource (Ågren et al., 2012; Bloom et al., 1985; Chapin et al., 1987). However, microbial and plant processes may be limited in their capacity to promote the acquisition of nutrients (Vitousek et al., 2010) such that nutritional balance cannot always be achieved. For example, although greater enzyme activity can increase the availability of a limiting nutrient, such as P (Marklein & Houlton, 2012; McGill & Cole, 1981; Olander & Vitousek, 2000), the effects are constrained by the availability of organic P substrate (German et al., 2011). Likewise, N fixation can balance N availability with that of P, but only within the constraints of temperature and carbon limitations of the process (Houlton et al., 2008; Vitousek et al., 2010). Moreover, the synchrony among nutrient cycles that contributes to co‐limitation takes time to develop and may be sensitive to environmental disturbances such as forest harvest that alter the recycling of one nutrient more than another (Rastetter et al., 2013).

Co‐limitation of primary productivity by N and P is widespread in marine, freshwater, and terrestrial ecosystems (Elser et al., 2007; Harpole et al., 2011; Jiang et al., 2019). Single‐nutrient limitation by N and by P has been detected in northern hardwood forest ecosystems (Finzi, 2009; Vadeboncoeur, 2010). However, experimental evidence of co‐limitation in these forests is scarce. Temperate hardwood forests were not well represented in the meta‐analyses showing co‐limitation, and those analyses found primarily P limitation, rather than co‐limitation, in the forest ecosystems that were included (Elser et al., 2007).

We established a study of Multiple Element Limitation in Northern Hardwood Ecosystems (MELNHE) to investigate nutrient limitation in mature and recently harvested forest stands across a wide range of site conditions. MELNHE is a long‐term, fully factorial N and P addition experiment conducted in central New Hampshire, USA, and the first of its kind in a temperate forest. The experiment includes stands that were classified at the beginning of the study in age categories that we refer to as young, mid‐age, or mature, reflecting the time since harvest and species composition at that time (2011). Our young stands (~21–28 years old in 2011) were dominated by early successional species. Our mid‐age stands (~31–41 years old) had experienced the transition in species composition in which pin cherry is lost, contributing high woody debris inputs, and *Betula papyrifera* Marsh. (white birch) increases in importance (Fahey et al., 1998). Forests in the region begin to more closely resemble the pre‐cut mature forest after around 50 years, with a mix of *Fagus grandifolia* Ehrh. (American beech), *Acer saccharum* Marsh. (sugar maple), and *B. alleghaniensis* Britton (yellow birch) (Marks & Bormann, 1972), although some white birch may persist for several more decades (Bormann et al., 1970).

Goswami et al. (2018) reported that tree diameter growth in the MELNHE study responded primarily to P over the first 4 years of treatment in 10 mid‐age and mature stands and to N in the three youngest stands; in some stands, the response to N + P exceeded that of the single nutrient that was most limiting, but not consistently enough to detect co‐limitation. Foliar nutrient concentrations (Hong et al., 2022) and resorption from senesced leaves (Gonzales et al., 2023; Gonzales & Yanai, 2019) supported the idea that P is more limiting than N. However, plant–soil feedbacks on nutrient availability were consistent with mechanisms supporting co‐limitation: Elevated availability of one nutrient suppressed litterfall recycling of the other nutrient (Goswami & Fisk, 2024), adding P caused N availability to decline in surface soils (Goswami & Fisk, 2024), and in mature forests, adding N caused P availability to decline (Shan et al., 2022). Fine root growth was co‐limited by N and P availability in several young (Li et al., 2024) and mid‐age (Butt et al., unpublished) forests, whereas in mature forests, fine root growth increased in response to N addition, but only without added P, suggesting an increase in allocation for P acquisition (Shan et al., 2022). These nutrient feedbacks take several years to develop fully, and the belowground responses to nutrients may not immediately benefit aboveground growth. Hence, Goswami et al. (2018) suggested that a general model of “sequential co‐limitation” may apply, in which N and P recycling are close to balanced, yet one of the co‐limiting nutrients is somewhat more limiting than the other at any one time (Craine, 2009;Davidson & Howarth, 2007; Elser et al., 2007). Alternatively, co‐limitation can be simultaneous if growth responds only to both nutrients together; additive, if the response to combined nutrients is similar to the sum of the response to individual nutrients; or synergistic, if the response to both nutrients combined is greater than expected from the sum of responses to single nutrients (Craine, 2009 ; Harpole et al., 2011). In nutrient addition experiments, the evidence for sequential limitation can develop over time as alleviating limitation by the first nutrient induces greater demand for the second. However, if both limitations are alleviated prior to observing responses, the sequential effects can be interpreted as synergistic (Elser et al., 2007).

We tested the hypothesis that aboveground tree growth in mature northern hardwood forests is sequentially co‐limited by P and N in a four‐year continuation of Goswami et al.'s (2018) study. We asked whether evidence of co‐limitation becomes more conclusive over this longer treatment period. The MEL model predicted that limitation in this ecosystem transitions from single limitation by N to single limitation by P around 30–35 years post harvest, before shifting to N‐P co‐limitation in mature forest (Rastetter et al., 2013). Therefore, we also tested the prediction that tree growth in our young and mid‐age stands is single‐nutrient limited, rather than co‐limited.

## METHODS

### Study site

**FIGURE 1:**
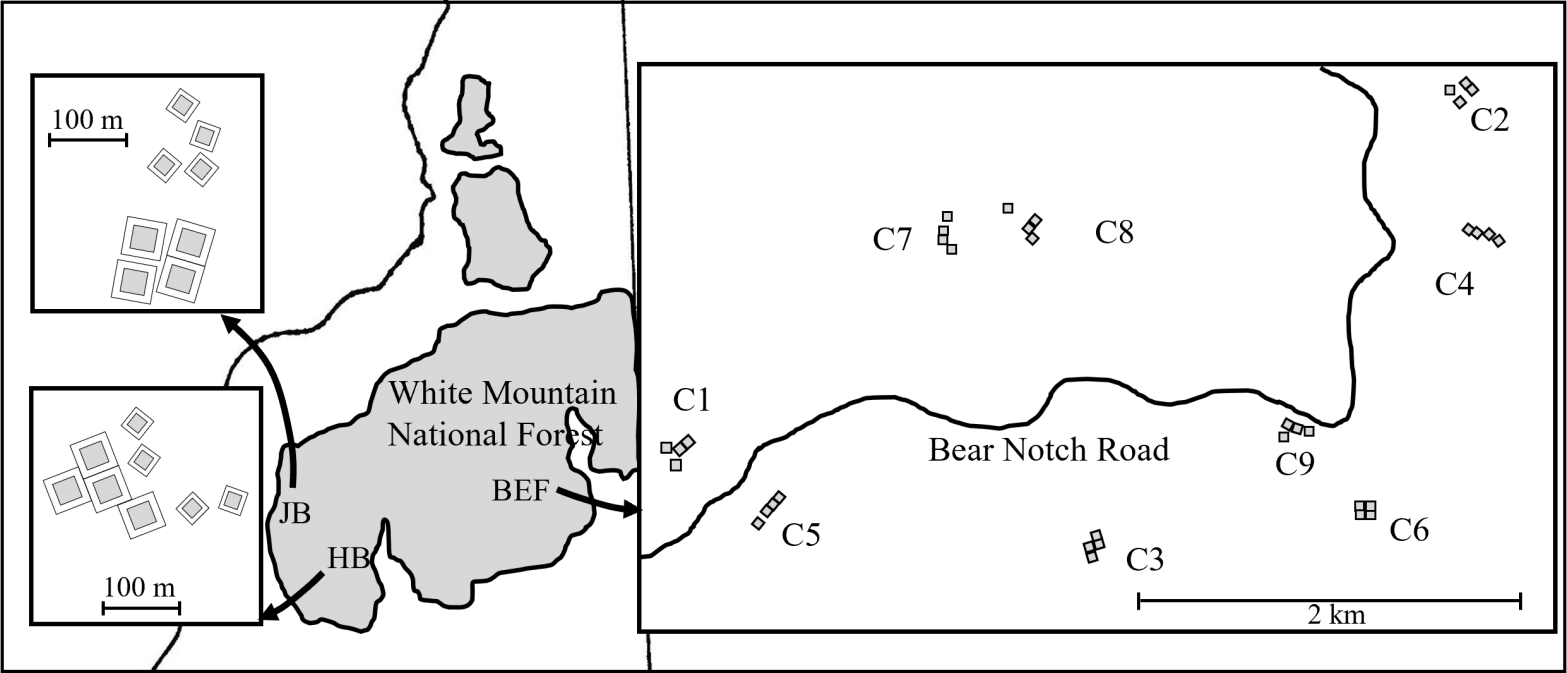
Map of study site locations in the White Mountain National Forest (shaded), New Hampshire (outlined), USA. Inner measurement areas and outer buffer zones are shown for HB and JB sites.

**FIGURE 2:**
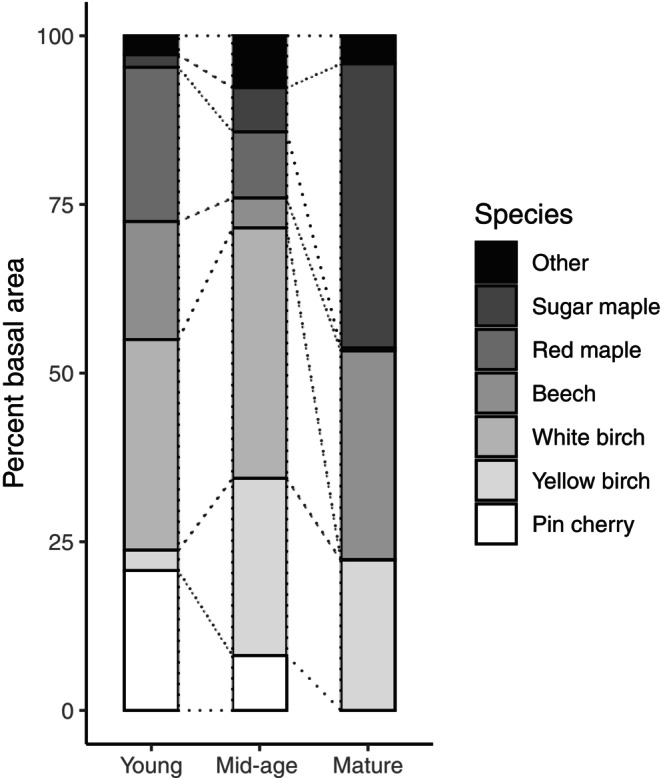
Pretreatment species composition of stems >10 cm dbh, by percent of total basal area in three age classes of northern hardwood forests: young (clearcut ~1983–1990), mid‐age (clearcut 1970–1980), and mature (>100 years old). Bars are means of 3 (young) or 5 (mid‐age and mature) stands.

The MELNHE study is an ongoing N and P addition experiment conducted in 13 stands located in Hubbard Brook Experimental Forest, Bartlett Experimental Forest, and Jeffers Brook, in central NH, USA (Figure 1). Stands were classified in age categories that we refer to as young (clearcut between ~1982 and 1990), mid‐age (clearcut between 1970 and 1980), or mature (heavily cutover before 1915; Table 1). When our study began (2011), the overstory in young stands was dominated by *Prunus pensylvanica* L.f. (pin cherry), *Acer rubrum* L. (red maple), *Fagus grandifolia* Ehrh. (American beech), and *B. papyrifera* Marsh. (white birch), whereas the mid‐age stands were dominated by *B. papyrifera* and *B. alleghaniensis* Britton (yellow birch; Figure 2; Appendix S1: Table S1). Mature forest overstory consists of *A. saccharum* Marsh. (sugar maple), *F. grandifolia*, and *B. alleghaniensis* (Figure 2). Of the nine stands at Bartlett, three stands each are classified as young, mid‐age, and mature. Hubbard Brook and Jeffers Brook each have one mid‐age stand and one mature stand (Table 1; Figure 1).

**TABLE 1:** Characteristics of 13 northern hardwood forest stands in the Bartlett Experimental Forest (C1–C9), Hubbard Brook Experimental Forest (HBM and HBO), and Jeffers Brook (JBM and JBO) in central New Hampshire, USA (including soil data summarized from Goswami & Fisk, 2024; Ratliff & Fisk, 2016).

Stand	Age class	Year clearcut	Elevation (m)	Mineral soil				Forest floor (Oe + Oa)		
				pH	Extractable Ca (μg g^−1^)	Extractable Al (μg g^−1^)	Extractable Fe (μg g^−1^)	Total C (g m^−2^)	N_min_ (mg m^−2^ day^−1^)	N_itr_ (% of N_min_)
C1	Young	1990	570	4.44	214	207	16	2696	82	0.1
C2	Young	1988	340	4.44	88	263	25	2506	29	0.6
C3	Young	~1982–1985	590	4.14	124	291	30	3336	118	16.6
C4	Mid‐age	1979	410	4.14	89	276	24	2933	29	0.4
C5	Mid‐age	1976	550	4.38	133	197	21	2403	75	3.6
C6	Mid‐age	1975	460	4.28	98	336	27	2647	76	9.9
C7	Mature	1890	440	4.75	72	222	12	1809	42	2.3
C8	Mature	1883	330	4.67	92	184	10	1886	27	0.0
C9	Mature	1890	440	4.65	89	218	10	2172	48	8.4
HBM	Mid‐age	1970	500	3.85	77	365	35	1700	73	36.5
HBO	Mature	1911	500	3.86	135	510	106	3220	73	21.2
JBM	Mid‐age	~1975	730	4.85	636	219	35	1540	33	80.4
JBO	Mature	1915	730	4.56	391	205	12	1642	74	68.5

*Note*: pH and extractable cations were measured in the upper 10 cm of the mineral soil. Nmin = net N mineralization potential, Nitr = net nitrification potential.

Soils are Typic and Aquic Haplorthods that formed following glacial retreat approximately 14,000 years ago, overlying amphibolite bedrock at Jeffers Brook and a variety of base‐poor metamorphic and igneous rocks at Hubbard Brook and Bartlett. Soils have surface organic horizons of 4–8 cm thickness and 1.54–3.34 kg C m^−2^, low pH ranging from 3.8 to 4.8 in mineral soils, and wide variation in extractable cation concentrations (Table 1). Soil conditions are described in detail by Vadeboncoeur et al. (2012, 2014). The regional climate is humid continental, with an average annual temperature at Hubbard Brook of 6°C and annual precipitation of 140 cm. Compared to the 1901–2000 mean, summers (June–August) during our study were on average 0.71°C warmer with 7.24 cm more precipitation at Bartlett Experimental Forest and 0.81°C warmer with 6.37 cm more precipitation at Hubbard Brook (NOAA, 2024). Average summer temperatures did not differ between the first and second measurement periods in our study (18.1°). However, greater summer precipitation during the first measurement period (40.7 cm) than the second (34.8 cm; NOAA, 2024) may have influenced tree growth rates.

### Treatment and data collection

Within a several‐hectare area in each stand, four 50 m × 50 m plots were established except in the Hubbard Brook and Jeffers Brook mid‐age stands, where plots were 30 m × 30 m. Plots were randomly assigned one of four fertilizer treatments: control, N, P, or N + P. To minimize the chance of N movement from one plot to another, we assigned N and N + P treatments downhill from others if plots were on a slope. Phosphorus is retained strongly in these soils and is unlikely to be mobile (Wood et al., 1984). All measurements were made in the interior 30 m × 30 m of each plot (20 m × 20 m in the smaller plots). Nitrogen was added as pelletized NH_4_NO_3_ (30 kg N ha^−1^ year^−1^) and P was added as powdered or granular NaH_2_PO_4_ (10 kg P ha^−1^ year^−1^) at the beginning of June of each year of the study. These relatively modest rates are intended to alter site fertility and maximize nutrient retention over the long term while minimizing acute artifacts associated with high doses of fertilizer. Fertilization increased in situ resin‐available N and P, and nitrification rates increased in response to N addition, especially in combination with P (Goswami & Fisk, 2024). After 10 years of treatment, mineral soil pH averaged 1.5% lower in plots receiving N alone (4.21) and 4% greater in plots receiving P alone (4.43) relative to controls (4.28), but 3% lower in plots receiving N + P together (4.14) (Fisk, 2022; *p* = 0.01 for the NxP interaction in two‐way ANOVA).

Trees ≥10 cm dbh were tagged and identified to species, and diameters of tagged trees were recorded in August 2011, 2015, and 2019 (Fisk et al., 2025). Beech bark disease affects measured diameters by making diameters larger in more diseased trees; beech leaf disease had not yet arrived in our stands in 2019.

We calculated relative basal area increment (RBAI) as an index of growth rate for each individual tree, for the measurement periods 2011–2015 and 2015–2019. We annualized the RBAI of each living tree as (1 + ((BA_F_ − BA_I_)/BA_I_))^1/*n*^ − 1, where BA_I_ and BA_F_ refer to individual tree basal area at the beginning and end, respectively, of the interval of *n* years. We also calculated total basal area increment in both measurement periods, for an ecosystem‐scale measure of tree growth, as the difference in the summed basal area of all the trees between measurement dates, divided by the time between measurements. In this case, we subtracted the living basal area in the first measurement from the living plus dead basal area in the second measurement to account for mortality.

We calculated density‐adjusted basal area increments by multiplying the basal area of each tree by its species‐specific wood density (Nowak, 2024) prior to summing basal area at the plot level to provide a metric more closely related to forest biomass than unweighted basal area. We also estimated the stand density index from stem numbers, diameters, and species‐specific wood density of each species, using equations of Ducey and Knapp (2010). Stand density affects tree allometry and biomass accumulation relative to tree volume in mixed‐species forest, and this index is intended to indicate biomass accumulation potential (Ducey & Knapp, 2010; Woodall et al., 2015).

### Data analysis

We evaluated treatment effects on forest growth using Bayesian linear mixed models of mean tree RBAI per plot, plot‐level basal area increment, and plot‐level density‐adjusted basal area increment (Stevens et al., 2025). These included main effects of N and P in a two‐way factorial design that can test the N × P interaction. The models also included stand age class, measurement period, and all associated interactions as fixed effects. We included stand as a random effect in a split plot design with repeated measurements for the two periods of 2011–2015 and 2015–2019. We transformed each response variable (RBAI, BA, density‐weighted BA) to the ¾ power to best achieve homoscedastic and normally distributed errors.

We used Hamiltonian Markov chain Monte Carlo (MCMC) with Stan to fit our Bayesian models and estimate all parameters, using the R programming language and environment (R Core Team, 2024), the rstanarm package in R, v. 2.32.1 (Goodrich et al., 2024), and the up‐to‐date versions of other packages upon which rstanarm depends (e.g., Rcpp v. 1.0.12, Eddelbuettel et al., 2024). Markov chains were run with 10,000 iterations with no thinning in each of four chains, half of which were warm‐up, resulting in a total of 20,000 MCMC samples. Our Bayesian priors were weakly informative, and reasonable variation in our priors did not alter the qualitative outcome of the analysis.

Our MCMC diagnostics included checking MCMC chain convergence and coverage using visual inspection of the chains with trace plots, autocorrelation plots, and rank ECDF plots (Säilynoja et al., 2022). We also inspected the posterior predictive distributions, quantitative estimates of $\hat{R}$, and effective sample sizes. All $\hat{R}$ were <1.001, indicating convergence of chains. Effective sample sizes ranged from 4700 to 15,000, indicating coverage of distributions sufficient for reliable interval estimates. We also checked visually for the appropriateness of the priors using the prior predictive distribution and the resulting fixed effects and SDs. These proved to be reasonable with ranges spanning plausible effect sizes.

We used model selection as one approach to estimate the strength of evidence for which factors best predict tree growth rates and basal area increments (Yates et al., 2023). The full (most complex) model included all possible interactions among all four fixed effects (N, P, age class, and measurement period). Model selection compared the full model with simpler models via approximate leave‐one‐out cross validation, using the loo package (Vehtari et al., 2023) to estimate the expected posterior log‐probability density (EPLD), which identifies the most parsimonious model. We compare the 10 most parsimonious models in Appendix S1: Tables S2–S4.

We estimated the effects of N and P on annual growth in a second approach using treatment contrasts. For this approach, we used the full model, rather than the most parsimonious model, because it retains all of the uncertainty necessary for credible intervals (Yates et al., 2023). All estimates were based on the expected posterior predictive distributions, a Bayesian equivalent of the expected marginal means. Contrasts based on the posterior predictive distributions and all 90% credible intervals (equal to 95% intervals on each tail of the distribution) were estimated using the emmeans package (v. 1.10.0; Lenth, 2024). We back‐transformed these estimates to present results as RBAI or basal area increment. Post‐processing of results and graphics was made using the up‐to‐date tidyverse suite of R packages (Wickham et al., 2019) and the tidybayes package (v. 3.0.6, Kay, 2023).

## RESULTS

### Stand age class comparisons

**FIGURE 3:**
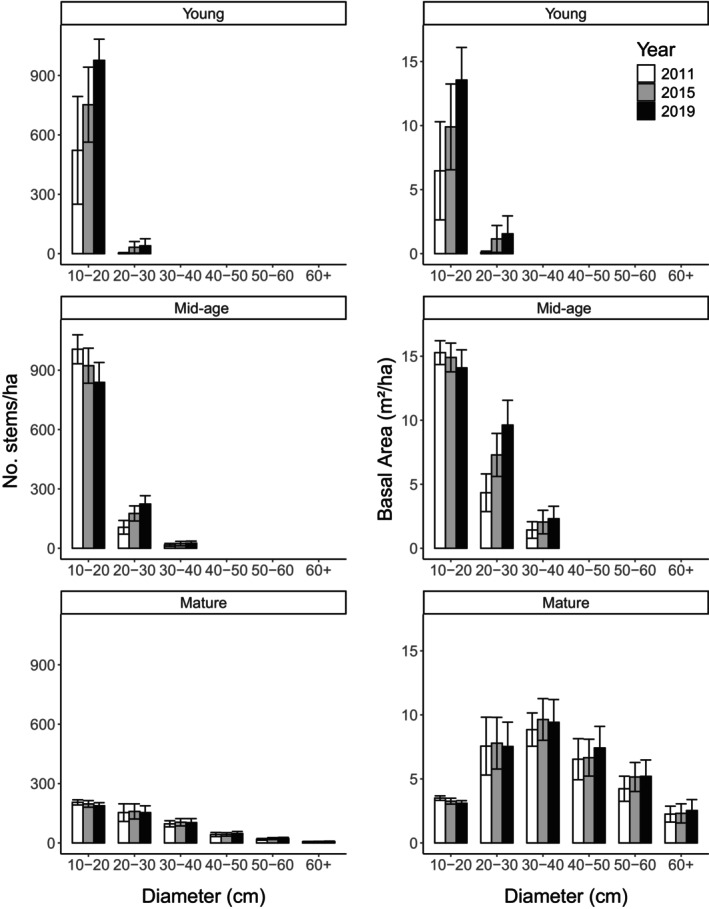
Stem density and basal area by diameter class in three age classes: young (clearcut approximately 1983–1990), mid‐age (clearcut 1970–1980), and mature (>100 years old). Means and SEs of the mean of 3 (young) or 5 (mid‐age and mature).

Basal area and stem densities were stable over the period of our study in mature stands but changed in young and mid‐age stands (Figure 3). For trees >10 cm diameter, basal area was greatest (36 m^2^ ha^−1^) and stem densities were lowest (560 ha^−1^) in mature stands. Basal area increased over time in successional stands, from 7 to 16 m^2^ ha^−1^ in young stands, mostly in trees <20 cm diameter, and from 22 to 27 m^2^ ha^−1^ in mid‐age stands, more in trees 20–30 cm diameter (Figure 3). Stem densities were greatest in mid‐age stands and declined over time, from 1280 to 950 stems ha^−1^, losing primarily trees <20 cm diameter. In contrast, in young stands stem densities increased from 550 to 1110 ha^−1^, mainly as a consequence of ingrowth into the smaller diameter class (Figure 3).

Individual tree growth (RBAI), averaged over the 8‐year study period, was greatest in young stands (6.4% year^−1^), intermediate in mid‐age (3.4% year^−1^), and lowest in mature stands (1.6% year^−1^), and total basal area increment was greatest in young (1.2 m^2^ ha^−1^ year^−1^), less in mid‐age (0.9 m^2^ ha^−1^ year^−1^), and lowest in mature stands (0.4 m^2^ ha^−1^). Age‐class differences in diameter growth rates and stand‐level basal area increments (Appendix S1: Figure S1) were consistent with average stand density indices of 0.53 in young, 0.81 in mid‐age, and 0.91 in mature stands in 2019, which indicate the greatest potential for further biomass accumulation in young stands and least in mature stands.

### Treatment responses

Model selection procedures identify robust models that can best predict new observations. We found the strongest support for the same model across all three response variables. This model included independent effects of N, P, stand age class, measurement period, the age × measurement period interaction, and stand as a random effect (Appendix S1: Tables S2–S4). For each response, model selection provided weak support for potential two‐way interactions between N and P, stand age, measurement period, as well as P × measurement period (Appendix S1: Tables S2–S4). Nonetheless, our model selection indicated the strongest support for a model in which tree growth response to nutrients was consistent through time.

**FIGURE 4:**
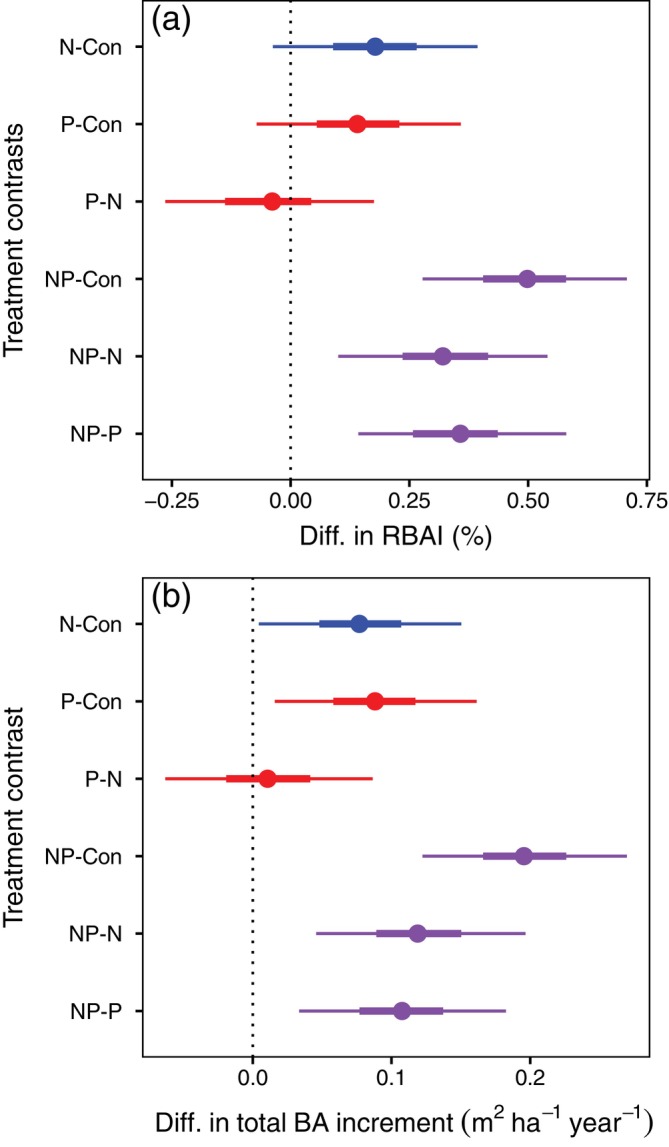
Contrasts showing responses to nutrient treatments as the difference (Diff.) in (a) relative basal area increment (RBAI) or (b) total basal area increment between N, P, or N + P treatments and controls, or between N + P and N or P treatments or controls. Intervals include the 50% and 90% credible intervals of differences between nutrient additions in expected (a) relative basal area increment (% year^−1^) and (b) total basal area increment (m^2^ ha^−1^ year^−1^). Differences have been backtransformed from estimates derived from posterior predictive distributions. Points represent the median of the interval of those differences. Colors represent the primary treatment for the contrast: N is blue, P is red, and N + P is purple. 0 on the *x*‐axis is marked by a dotted line.

Tree growth was greatest in plots receiving N + P across all stand ages and measurement periods (Table 2), indicating co‐limitation by N and P. Plot‐level basal area increment more clearly increased in response to additions of N and P alone than did RBAI, which was less consistent across stands (Figure 4). RBAI of trees and total basal area increment of plots responded more to N + P than to N or P alone (NP‐P, NP‐N, and NP‐Con contrasts are greater than zero), and responses to N and P alone were similar to each other (Figure 4). This response to N + P was additive, not synergistic; treatment contrasts do not show that the effects of N and P together are greater than expected by the effects of N and P alone (Figure 4). We found no evidence that the response to nutrients differed among stand age classes.

**TABLE 2:** RBAI and basal area increment (2011–2019) in three age classes of northern hardwood forests: young (clearcut ~1983–1990), mid‐age (clearcut 1970–1980), and mature (>100 years old).

Age class	Treatment			
	Con	N	P	NP
RBAI (% year^−1^)				
Young	6.3 (0.88)	6.4 (0.78)	6.1 (0.57)	6.9 (1.10)
Mid‐age	3.1 (1.38)	3.5 (1.56)	3.5 (1.54)	3.7 (1.66)
Mature	1.4 (0.18)	1.4 (0.06)	1.7 (0.23)	1.8 (0.16)
Basal area increment (m^2^ ha^−1^ year^−1^)				
Young	1.03 (0.031)	1.18 (0.116)	1.17 (0.056)	1.37 (0.123)
Mid‐age	0.83 (0.028)	0.95 (0.116)	0.91 (0.057)	1.00 (0.013)
Mature	0.40 (0.040)	0.40 (0.047)	0.45 (0.036)	0.50 (0.033)

*Note*: SEs of the mean are in parentheses; *n* = 3 (young) or 5 (mid‐age and mature) stands.

Abbreviation: RBAI, relative basal area increment.

Responses by wood‐density adjusted basal area increment were very similar to those of basal area increment, except that basal area increment in the N treatment differed more from that of the control (for the density‐adjusted metric, the N–Control contrast overlaps with zero; Figure 4 and Appendix S1: Figure S2). The similarity of the response of the wood‐density adjusted basal area improves our confidence that the diameter growth results we report are relevant to forest biomass in spite of differences in wood density across species.

We evaluated the possibility of sequential co‐limitation, which would be indicated by differences between the two measurement periods in the co‐limitation contrasts of NP versus N or NP versus P treatments. However, for RBAI, the 90% credible interval for the difference between measurement periods is nearly centered on zero (−0.92 to 0.69% year^−1^ for the NP vs. N contrasts and −0.77 to 1.01% year^−1^ for the NP vs. P contrasts). Similarly, for total basal area increment, the 90% credible interval for the difference between measurement periods was −0.28 to 0.33 m^2^ ha^−1^ year^−1^ for the NP versus N contrasts and −0.24 to 0.34 m^2^ ha^−1^ year^−1^ for the NP versus P contrasts. Thus, we do not have evidence of the sequential development of co‐limitation over the first 8 years of treatment, although the evidence for additive co‐limitation is clear.

## DISCUSSION

After only four years of treatment, we reported greater limitation by P than by N in this study system (Goswami et al., 2018). An additional tree inventory conducted four years later, together with marked ingrowth in the youngest stands, improved our capacity to test for tree growth co‐limitation in the MELNHE study. We found clear co‐limitation by N and P in our 13 study sites; the growth response to N and P together was greater than the response to either nutrient alone (Figure 4). Additive co‐limitation was indicated by the response of basal area increment to N and to P, individually, with no apparent interaction between these main effects. Adjusting basal area increments by species‐specific wood density suggests the same treatment responses for the biomass of wood production.

Our results are consistent with meta‐analyses that showed the prevalence of co‐limitation by N and P in a variety of terrestrial and aquatic ecosystems (Elser et al., 2007; Harpole et al., 2011; Jiang et al., 2019), but it is notable that forested ecosystems in Elser et al.'s (2007) analysis tended to be P limited rather than co‐limited. Tests of co‐limitation are not common in north temperate forests (Vadeboncoeur, 2010), and the MELNHE study complements other work to extend the breadth of ecosystem types in which co‐limitation has been shown. It also complements belowground work in the MELNHE stands to improve our understanding of whole‐tree limitation in this ecosystem. Fine root growth in young‐ and mid‐successional MELNHE stands has also shown co‐limitation by N and P (Li et al., 2024; Butt et al., unpublished), but in mature stands, Shan et al. (2022) found N limitation of fine root growth and proposed that this contributes to co‐limitation by improving P acquisition if N availability is high.

Our results indicate that co‐limitation by N and P can be sustained across forest stands that vary widely in N status and that differ in successional stage. That we are able to detect co‐limitation across the range of N status represented in our 13 stands is consistent with evidence of the coupling between N and P cycling across MELNHE stands (Ratliff & Fisk, 2016; See et al., 2015). Forest harvest is likely to disrupt that coupling and disproportionately affect N and P recycling, because of high N losses following large‐scale disturbances (Vitousek & Melillo, 1979) and low N:P ratios in woody residues (Whittaker et al., 1979) that may increase immobilization of N more than that of P early in forest regeneration (Rastetter et al., 2013). Simulations using the Multiple Element Limitation (MEL) model suggest that harvest disrupts the balance of N and P recycling to cause limitation that shifts from N (15–25 years after harvest) to P (30–80 years) and finally to co‐limitation in mature forests as N and P cycles resynchronize (Rastetter et al., 2013). However, we did not find a change from single‐nutrient limitation in young forests to co‐limitation in mature forests: In all of the age classes that we studied, forest growth was co‐limited by N and P.

Our results do not clearly support the idea of sequential co‐limitation, in which one nutrient is transiently more limiting than the other, while over time N and P together are limiting (Craine, 2009). Co‐limitation might be expected to oscillate between N and P, as the response to the nutrient in slightly greater supply induces greater demand for the other (Davidson & Howarth, 2007). This oscillation can be rapid, as in aquatic systems, and if the initial shift from one nutrient to the other is measured together, then the outcome appears to be synergistic (Elser et al., 2007). In trees, increased nutrient uptake into foliage can be rapid in response to fertilization, and oscillations may occur rapidly at biochemical or physiological levels. However, ecosystem adjustments in belowground carbon allocation could be much slower, involving altered root biomass or mycorrhizal associations and feedback in the rates of immobilization and mineralization during decay. Thus, sequential co‐limitation in forests might be expected to take many years to detect.

After the first four years of treatment in the MELNHE study, co‐limitation of individual tree growth (RBAI) was suggested but not statistically significant in several of our stands (Goswami et al., 2018). The development of N limitation in P‐fertilized plots, indicated by lower litterfall N recycling and resin‐available N (Goswami & Fisk, 2024), could occur under sequential co‐limitation. However, we did not find a progression of tree growth response to P and then to N + P addition between the first and second 4‐year measurement intervals in our current analysis. In addition, sequential co‐limitation would not predict our observation of plot‐level basal area responses to both N and P singly (Craine, 2009). Instead, adding a second measurement period provided better support for additive co‐limitation of individual tree growth over the entire eight‐year study period, probably because of improved statistical power to detect responses.

We contend that N‐P co‐limitation of forest growth is likely widespread across northern hardwood forests in our region, but this may change in response to ongoing environmental change. A key driver may be N oligotrophication (Groffman et al., 2018; Mason et al., 2022) due to CO_2_ fertilization and declining atmospheric N deposition (Ackerman et al., 2019; Lloret & Valiela, 2016). Declining N availability could shift forests from N‐P co‐limitation toward single‐nutrient limitation by N. However, co‐limitation could be maintained if the processes driving N oligotrophication cause proportionate declines in P availability, through biomass or soil organic matter sequestration, or if compensating mechanisms constrain P availability. For example, recycling or weathering of P could decrease if reduced N supply limits enzymatic activity. Soil de‐acidification accompanying declining atmospheric inputs of strong acids could further alter soil P availability. Finally, increasing temperature and precipitation also have uncertain implications for N‐P co‐limitation. Continued monitoring will reveal whether imbalance develops in the availability of N relative to P, in which case the persistence of co‐limitation will depend on whether plant allocation responses and plant–soil feedbacks contributing to co‐limitation respond as effectively as the processes that alter N availability relative to P.

In summary, following long‐term nutrient treatments in a northern hardwood forest, we found evidence that tree and forest growth are co‐limited by N and P. Co‐limitation was consistent among successional stages, suggesting that it can develop long before forests reach a steady state with respect to biomass. Our findings in north temperate forests contribute to the growing body of evidence for co‐limitation by N and P globally (Elser et al., 2007). A more comprehensive understanding of the mechanisms sustaining nutrient co‐limitation may emerge as forests respond to shifting environmental conditions—particularly the ongoing decline in atmospheric N deposition and the rising influence of elevated atmospheric CO₂ levels. These changes have the potential to alter nutrient demand, uptake efficiency, and allocation patterns within forest ecosystems, thereby testing the effectiveness of the processes that currently promote co‐limitation of N and P.

## CONFLICT OF INTEREST STATEMENT

The authors declare no conflicts of interest.

## Supporting information


Appendix S1:

## Data Availability

Data (Fisk, 2022; Fisk et al., 2025; Stevens et al., 2025) are available in the Environmental Data Initiative repository at https://doi.org/10.6073/pasta/fb8f8d5b903627bee9ad6aa4c32f2289, https://doi.org/10.6073/pasta/275ad28a2f31356cf9c2648531a16a2b, and https://doi.org/10.6073/pasta/83dfe28aeb72ab797319847a17b2bc85, respectively.
