# Supplementary material for: Nitrogen and phosphorus co‐limitation of tree growth in northern hardwood forests
Source: Ecology. 2025 Oct 7;106(10):e70217. doi: 10.1002/ecy.70217 (PMC12501703; doi:10.1002/ecy.70217)
Supplement: Supplementary file 1 — Appendix S1: [file ECY-106-e70217-s001.pdf]

Appendix S1

Nitrogen and phosphorus co-limitation of tree growth in northern hardwood forests

Noah M. Blumenthal, M. Henry H. Stevens, Shinjini Goswami, Ruth D. Yanai, Timothy J. Fahey, Melany C. Fisk

Ecology

Supplementary Tables

Table S1. Fraction of total basal area in treatment plots in each stand, in 2011 (first treatment year) and 2019. Acru= *Acer rubrum*, Acsa = *A. saccharum*, Beal = *Betula alleghaniensis*, Bepa = *B. papyrifera*, Fagr = *Fagus grandifolia*, Prpe = *Prunus pensylvanica*, Populus = *Populus grandidentata* plus *P. tremuloides*. Other includes *Abies balsamifera, Acer pensylvanicum, Fraxinus americana, Picea rubens, Prunus serotina, Quercus rubra, Sorbus americana, Tilia americana,* and *Tsuga canadensis.*

		2011				2019			
Stand	Species	Con	N	P	NP	Con	N	P	NP
					Young stands				
C1	Acru	0	0	0	4.0	0	0	0	1.5
	Acsa	0	0	0	0	1.7	0	0	0
	Beal	0	0	0	0	5.2	2.4	1.4	4.5
	Bepa	61.8	52.2	56.8	76.2	67.9	64.5	63.6	71.6
	Fagr	29.5	0	7.4	0	15.2	2.3	2.9	1.5
	Prpe	8.7	47.8	35.9	14.6	7.9	24.2	27.2	19.8
	other	0	0	0	5.1	2.2	6.5	4.9	1.1
C2	Acru	54.5	44.0	30.8	62.1	42.5	35.4	32.0	32.5
	Acsa	0	0	0	0	0	0	0	0
	Beal	0	0	5.1	0	2.3	0.8	5.4	2.0
	Bepa	23.0	24.6	9.6	13.7	22.5	29.4	20.6	17.1
	Fagr	3.5	22.3	33.9	18.0	10.9	25.1	24.2	43.5
	Prpe	11.4	4.6	18.7	6.2	19.1	5.5	12.1	4.0
	other	7.6	4.5	2	0	2.8	3.9	5.8	0.9

Table S1, continued.

C3	Acru	4.5	21.9	16.4	36.1	5.2	23.9	16.2	37.3
	Acsa	1.1	1.9	4.3	14.9	2.2	1.7	5.8	13.0
	Beal	11.7	5.1	10.5	3.8	17.6	7.9	9.3	4.8
	Bepa	16.0	13.3	20.5	6.1	16.2	12.7	18.7	6.4
	Fagr	23.6	22.1	28.0	22.2	34.5	36.0	32.8	27.3
	Prpe	40.7	29.7	18.8	12.1	20.0	11.8	13.9	5.6
	other	2.4	5.9	1.5	4.9	4.3	6.0	3.3	2.0
Mid-age stands									
C4	Acru	17.7	0.6	2.0	8.9	22.2	1.3	2.7	13.4
	Acsa	0.6	0	0	0	0.6	0	0	0
	Beal	0.5	4.8	6.1	1.4	1.3	5.7	8.6	1.7
	Bepa	58.3	17.8	64.9	35.7	56.8	15.1	61.8	32.8
	Fagr	3.9	1.7	3.4	6.3	6.2	3.8	6.8	10.5
	Populus	1.4	70.2	0	30.8	1.3	71.5	0	33.7
	Prpe	8.5	3.2	5.5	13.3	0.9	0	0.7	4.3
	other	9.0	1.7	18.1	3.5	10.7	2.6	19.4	3.5
C5	Acru	10.7	4.4	3.7	4.1	12.1	5.5	4.9	4.4
	Acsa	1.0	1.7	0.7	0.8	1.5	2.9	1.6	1.7
	Beal	6.2	0.6	4.8	6.1	6.4	2.0	5.9	0.6
	Bepa	66.1	81.7	77.5	79.4	63.3	79.0	78.7	81.7
	Fagr	4.3	5.1	2.6	3.6	8.8	8.6	4.0	5.1
	Populus	0	0	1.8	2.2	0	0	2.1	0
	Prpe	8.8	6.5	8.9	3.3	4.2	1.0	1.5	6.5
	other	2.9	0	0	0.5	3.7	1.0	1.4	0
C6	Acru	18.8	31.9	23.8	23.5	22.2	36.6	24.8	31.9
	Acsa	6.4	2.0	2.3	2.2	7.4	2.1	2.3	2.0
	Beal	31.8	24.4	18.5	20.2	29.4	24.7	20.0	24.4
	Bepa	22.3	19.2	29.1	30.2	22.5	19.1	31.0	19.2
	Fagr	7.9	11.5	14.5	16.5	9.1	13.2	17.6	11.5
	Populus	0	1.7	2.1	2.3	0	1.5	2.4	1.7
	Prpe	2.7	7.4	7.6	3.2	0	1.2	0.5	7.4
	other	10.2	1.8	2.0	1.9	9.5	1.6	1.4	1.8
HBM	Acru	9.8	9.3	8.9	10.2	10.2	9.7	11.7	9.3
	Acsa	10.9	16.2	7.8	8.4	9.5	15.6	9.1	16.2
	Beal	49.6	33.6	36.8	41.7	54.6	39.9	42.7	33.6
	Bepa	16.8	9.3	25.0	24.4	15.4	9.0	25.4	9.3
	Fagr	1.0	0	4.5	5.6	0.9	0	6.9	0
	Populus	10.5	19.3	0	0	9.3	13.6	0	19.3
	Prpe	1.4	0	12.8	5.1	0	0	0	0
	other	0	12	4.2	4.6	0	12.2	3.5	12.3

Table S1, continued.

JBM	Acru	0	0	0	0	0	0	0	0
	Acsa	25.3	1.9	8.9	9.7	24.0	3.4	11.3	1.9
	Beal	30.9	45.7	37.2	41.3	36.1	68.2	44.3	45.7
	Bepa	16.6	31.0	23.5	23.7	15.8	27.1	18.6	31.0
	Fagr	2.2	0	0	0	1.9	0	0.8	0
	Prpe	4.9	21.4	13.1	7.1	0	1.3	3.7	21.4
	Populus	20.1	0	14.1	15.1	22.2	0	17.7	0
	other	0	0	3.2	3.2	0	0	3.4	0
					Mature stands				
C7	Acru	0	0	3.3	0	0	0	0	0
	Acsa	40.7	34.1	34.4	36.9	41.9	34.7	36.3	34.1
	Beal	4.8	2.2	8.2	8.9	4.9	1.5	9.0	2.2
	Fagr	54.5	61.8	39.7	38.1	53.2	63.2	38.3	61.8
	other	0	1.9	14.4	16.1	0	0.6	16.4	1.9
C8	Acru	0	5.5	0	0	0	4.6	0	5.5
	Acsa	40.7	41.7	45.0	44.6	40.6	45.3	46.6	41.7
	Beal	0	18.7	1.5	1.7	0.0	16.4	1.9	18.7
	Fagr	44.8	32.1	39.7	40.5	44.7	31.1	37.8	32.1
	other	14.5	2.1	13.8	13.3	14.7	2.6	13.7	2.1
C9	Acru	0	0	0	0	0	0	0	0
	Acsa	50.4	39.1	71.1	70.0	45.8	44.3	70.0	39.1
	Beal	27.7	24.8	15.0	15.1	31.4	17.5	12.2	24.8
	Fagr	21.9	36.0	14.0	14.9	22.7	38.2	17.9	36.0
	other	0	0	0	0	0	0	0	0
HBO	Acru	0	0	0	0	0	0	0	0
	Acsa	6.9	18.3	1.1	1.1	17.1	17.6	1.1	18.3
	Beal	46.6	66.4	68.9	69.8	49.5	65.5	70.0	66.4
	Fagr	45.7	15.3	24.0	23.0	29.6	16.9	22.9	15.3
	other	0.8	0	6.0	6.1	3.8	0	6.0	0
JBO	Acru	0	0	0	0	0	0	0	0
	Acsa	100.0	69.8	78.7	79.8	100.0	70.2	83.2	69.8
	Beal	0	22.5	15.5	14.1	0	20.0	11.7	22.5
	Fagr	0	5.0	5.8	6.1	0	6.3	5.1	5.0
	other	0	2.8	0	0	0	3.6	0	2.8

Table S2. Tree growth models. Response variable *y* is mean RBAI raised to the ¾ power, to achieve homoscedastic and normally distributed errors. The factor *yr* is measurement period. The term (1 | stand) indicates a random effect of stand, such that each stand is adjusted by its own mean. Rel. Lik. - relative likelihood, LOO-IC Wt - leave-one-out information criterion weight relative to the other models presented here, *EPLD* is expected posterior log-probability density (larger indicates more support), and *Diff* is the difference between the most parsimonious model (row 1) and each other model. *SE Diff* is the standard error of the difference. Thus, models that differ from the best model by less than its standard error have some support.

Model	Rel. lik.	LOO-IC Wt	ELPD	Diff	SE Diff
y ~ age + N + P + yr + (1 | stand) + age:yr	1.00	0.23	351.5	0	0
y ~ age + N + P + yr + (1 | stand) + age:yr + N:yr	0.47	0.11	350.7	-0.7	0.97
y ~ age + N + P + yr + (1 | stand) + age:yr + N:P	0.47	0.11	350.7	-0.8	0.92
y ~ age + N + P + yr + (1 | stand) + age:yr + P:yr	0.44	0.10	350.7	-0.8	0.92
y ~ age + N + P + yr + (1 | stand) + age:N + age:yr	0.43	0.10	350.6	-0.8	1.79
y ~ age + N + P + yr + (1 | stand) + age:N + age:yr + N:P	0.21	0.05	349.9	-1.6	2.05
y ~ age + N + P + yr + (1 | stand) + age:yr + N:P + P:yr	0.20	0.05	349.9	-1.6	1.22
y ~ age + N + P + yr + (1 | stand) + age:N + age:yr + N:yr	0.20	0.05	349.9	-1.6	2.02
y ~ age + N + P + yr + (1 | stand) + age:N + age:yr + P:yr	0.19	0.04	349.8	-1.6	2.07
y ~ age + N + P + yr + (1 | stand) + age:yr + N:P + N:yr	0.19	0.04	349.8	-1.7	1.12

Table S3. Basal area increment models. Response variable *y* is total plot basal area raised to the ¾ power, to achieve homoscedastic and normally distributed errors. The factor *yr* is measurement period. The term (1 | stand) indicates a random effect of stand, such that each stand is adjusted by its own mean. Rel. Lik. - relative likelihood, LOO-IC Wt - leave-one-out information criterion weight relative to the other models presented here, *EPLD* is expected posterior log-probability density (larger indicates more support), and *Diff* is the difference between the most parsimonious model (row 1) and each other model. *SE Diff* is the standard error of the difference. Thus, models that differ from the best model by less than its standard error have some support.

Model	Rel. lik.	LOO-IC Wt	ELPD	Diff	SE Diff
y ~ age + N + P + yr + (1 | stand) + age:yr	1.00	0.21	61.2	0	0
y ~ age + N + P + yr + (1 | stand) + age:N + age:yr	0.69	0.15	60.8	-0.4	2.03
y ~ age + N + P + yr + (1 | stand) + age:yr + N:P	0.43	0.09	60.3	-0.8	0.55
y ~ age + N + P + yr + (1 | stand) + age:yr + N:yr	0.43	0.09	60.3	-0.8	1.05
y ~ age + N + P + yr + (1 | stand) + age:yr + P:yr	0.34	0.07	60.1	-1.1	0.39
y ~ age + N + P + yr + (1 | stand) + age:N + age:yr + N:yr	0.33	0.07	60	-1.1	2
y ~ age + N + P + yr + (1 | stand) + age:N + age:yr + P:yr	0.29	0.06	59.9	-1.2	2.24
y ~ age + N + P + yr + (1 | stand) + age:N + age:yr + N:P	0.25	0.05	59.8	-1.4	2.03
y ~ age + N + P + yr + (1 | stand) + age:yr + N:P + N:yr + P:yr + N:P:yr	0.17	0.04	59.4	-1.8	2.28
y ~ age + N + P + yr + (1 | stand) + age:P + age:yr	0.17	0.04	59.4	-1.8	1.11

Table S4. Wood-density adjusted basal area increment models. Response variable *y* is total plot basal area raised to the ¾ power, to achieve homoscedastic and normally distributed errors. The factor *yr* is measurement period. The term (1 | stand) indicates a random effect of stand, such that each stand is adjusted by its own mean. Rel. Lik. - relative likelihood, LOO-IC Wt - leave-one-out information criterion weight relative to the other models presented here, *EPLD* is expected posterior log-probability density (larger indicates more support), and *Diff* is the difference between the most parsimonious model (row 1) and each other model. *SE Diff* is the standard error of the difference. Thus, models that differ from the best model by less than its standard error have some support.

Model	Rel. lik.	LOO-IC Wt	ELPD	Diff.	SE Diff.
y ~ age + N + P + yr + (1 | stand) + age:yr	1.00	0.21	109.6	0	0
y ~ age + N + P + yr + (1 | stand) + age:N + age:yr	0.72	0.15	109.3	-0.3	1.99
y ~ age + N + P + yr + (1 | stand) + age:yr + N:P	0.45	0.09	108.8	-0.8	0.75
y ~ age + N + P + yr + (1 | stand) + age:yr + N:yr	0.42	0.09	108.7	-0.9	1.21
y ~ age + N + P + yr + (1 | stand) + age:yr + P:yr	0.33	0.07	108.5	-1.1	0.23
y ~ age + N + P + yr + (1 | stand) + age:N + age:yr + N:P	0.33	0.07	108.5	-1.1	2.13
y ~ age + N + P + yr + (1 | stand) + age:N + age:yr + P:yr	0.31	0.06	108.4	-1.2	2.21
y ~ age + N + P + yr + (1 | stand) + age:N + age:yr + N:yr	0.28	0.06	108.3	-1.3	2.03
y ~ age + N + P + yr + (1 | stand) + age:yr + N:P + N:yr + P:yr + N:P:yr	0.20	0.04	108	-1.6	2.29
y ~ age + N + P + yr + (1 | stand) + age:yr + N:P + N:yr	0.20	0.04	108	-1.6	1.23

Supplementary Figures

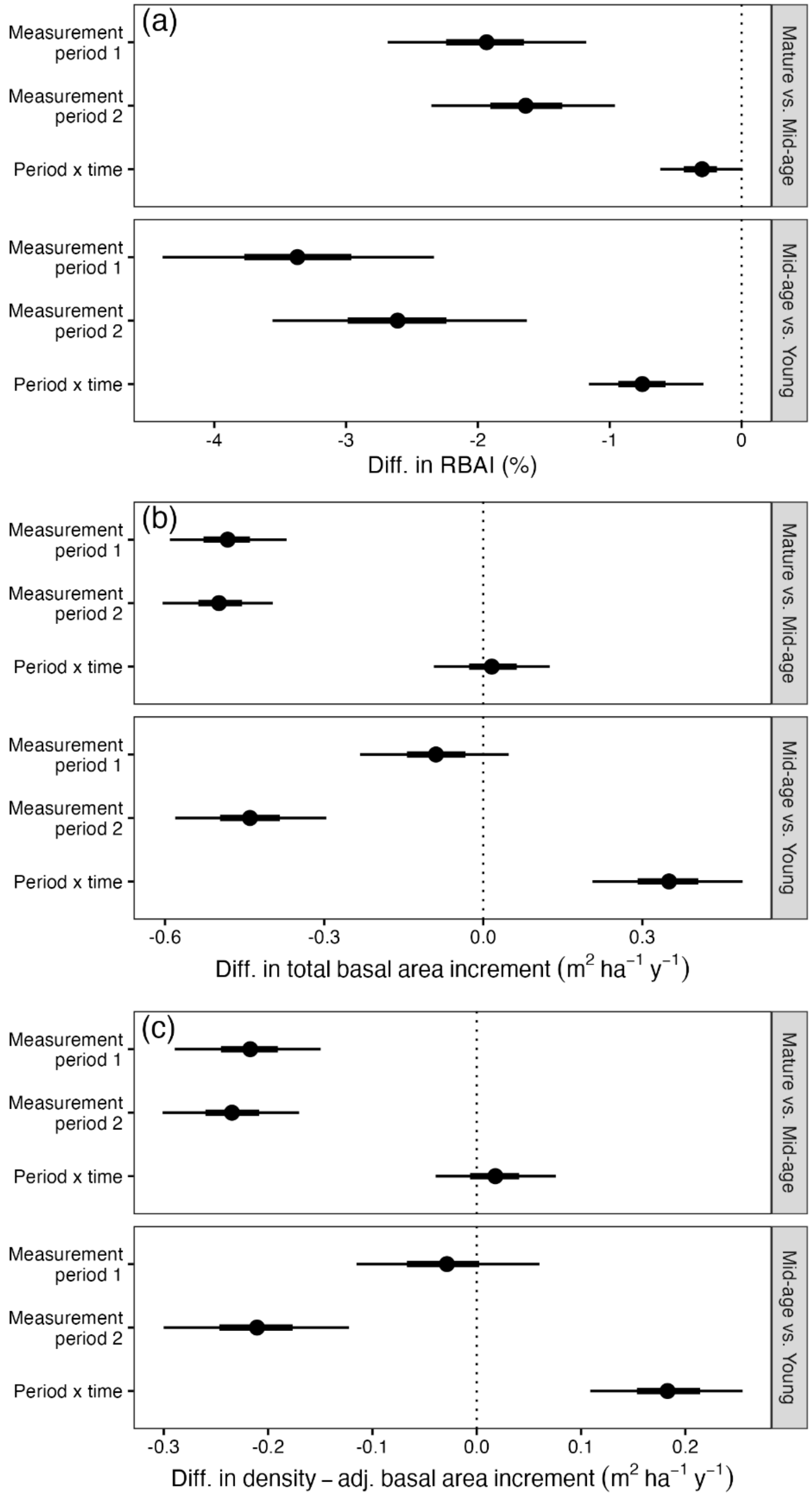

Figure S1: Contrasts showing differences (Diff.) between stand age classes (mature - mid-age;

mid-age - young) in (a) relative basal area increment (RBAI) of individual trees, (b) total basal area increment, and (c) density-adjusted (adj.) basal area increment. Differences between age classes are separated by measurement period (2011-2015; 2015-2019) to show age class x measurement period interactions, and are also contrasted between measurement periods (change in difference): for example the negative change in difference contrasts for RBAI indicate smaller differences between age classes in the second measurement period than the first. Intervals include the 50% and 90% credible intervals of differences between stand age classes and between measurement periods. Differences have been backtransformed from estimates derived from posterior predictive distributions. Points represent the median of the interval of those differences, either between age classes or measurement periods. 0 on the x-axis is marked by a dotted line.

In contrast to individual tree growth (a), the differences in basal area increment between mid-age and young stands were greater in the second measurement period (b and c), owing to greater ingrowth in young stands and higher mortality in the smallest stems in mid-age stands. Biomass differences will scale differently than basal area differences, with the larger trees in older stands contributing more biomass per unit basal area due to the exponential relationship between tree diameter and biomass (ie, Ter-Mikaelian and Korzuhkin 1997).

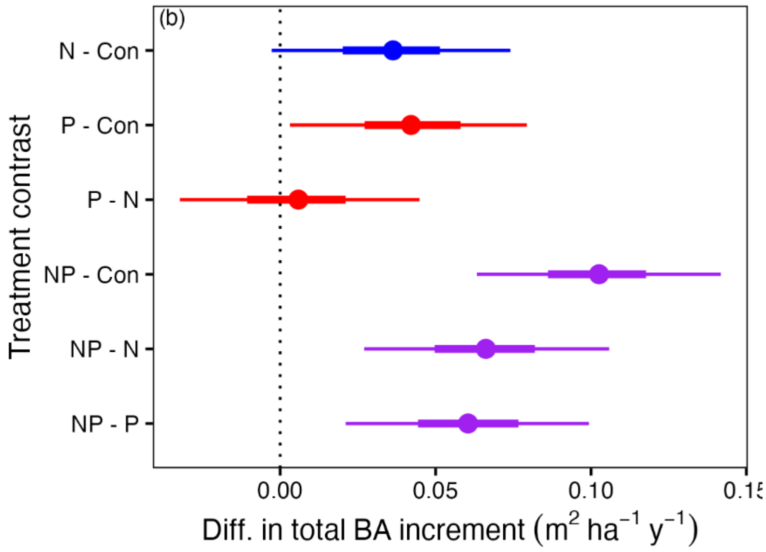

Figure S2: Contrasts showing responses to nutrient treatments as the difference (Diff.) in wood-density adjusted total basal area increment between N, P, or N+P treatments and controls, or between N+P and N or P treatments or controls. Intervals include the 50% and 90% credible intervals of differences between nutrient additions in expected wood-density adjusted total basal area increment (m^2 ha^{-1} yr^{-1}). Differences have been backtransformed from estimates derived from posterior predictive distributions. Points represent the median of the interval of those differences. Colors represent the primary treatment for the contrast: N is blue, P is red, and N+P is purple. 0 on the x-axis is marked by a dotted line.

Literature Cited

Ter-Mikaelian, Michael T., and Korzukhin, Michael D. 1997. Biomass equations for sixty-five North American tree species. Forest Ecology and Management 97: 1-24.
